# New record of *Nausithoe werneri* (Scyphozoa, Coronatae, Nausithoidae) from the Brazilian coast and a new synonymy for *Nausithoe maculata*

**DOI:** 10.3897/zookeys.984.56380

**Published:** 2020-11-04

**Authors:** Clarissa Garbi Molinari, Maximiliano Manuel Maronna, André Carrara Morandini

**Affiliations:** 1 Departamento de Zoologia, Instituto de Biociências, Universidade de São Paulo, Rua do Matão, travessa 14, n. 101, Cidade Universitária, São Paulo, SP, 05508-090, Brazil Universidade de São Paulo São Paulo Brazil; 2 Centro de Biologia Marinha, Universidade de São Paulo, Rodovia Manuel Hypólito do Rego km 131.5, São Sebastião, SP, 11600-000, Brazil Universidade de São Paulo São Paulo Brazil

**Keywords:** Coronamedusae, jellyfish, life cycle, periderm, polyp, scyphomedusae, systematics

## Abstract

The order Coronatae (Scyphozoa) includes six families, of which Nausithoidae Haeckel, 1880 is the most diverse with 26 species. Along the Brazilian coast, three species of the genus *Nausithoe* Kölliker, 1853 have been recorded: *Nausithoe atlantica* Broch, 1914, *Nausithoe punctata* Kölliker, 1853, and *Nausithoe aurea* Silveira & Morandini, 1997. Living polyps (*n* = 9) of an unidentified nausithoid were collected in September 2002 off Arraial do Cabo (Rio de Janeiro, southeastern Brazil) at a depth of 227 m, and have been kept in culture since then. We compared these specimens with three species cultured in our laboratory: *Nausithoe aurea* (from Ilhabela, São Paulo, Brazil), *Nausithoe maculata* Jarms, 1990 (from Cuba and Puerto Rico), and *Nausithoe werneri* Jarms, 1990 (from the Atlantic Ocean off Morocco and from the Mediterranean Sea). The criteria used for comparison were: main aspects of the morphology, life cycle, and DNA sequences (18S, 28S, and COI). The results indicate that the unidentified polyps belong to *N. werneri*. Furthermore, *N. aurea* is considered a junior synonym of *N. maculata*.

## Introduction

The class Scyphozoa Goette, 1887 (true jellyfishes) comprises two main clades: Coronamedusae Calder, 2009 and Discomedusae Haeckel, 1880 (Jarms and Morandini 2019). The Coronamedusae have a single class, Coronatae Vanhöffen, 1892, which is mostly known from the deep-sea medusae of the genera *Atolla* Haeckel, 1880, *Periphylla* Müller, 1861, and *Paraphyllina* Maas, 1903. In addition, the group also includes several shallow-water species in the genera *Nausithoe* Kölliker, 1853 and *Linuche* Eschscholtz, 1829 (Jarms and Morandini 2019). Coronate species are distinguished from other scyphomedusae by the presence of a coronal furrow (groove) on the exumbrella (Mayer 1910; Russell 1970). Of the 59 species of coronates, the life cycles of 16 have been described (Jarms 2010). All of these have a metagenetic life cycle, with the holoplanktonic *Periphylla periphylla* (Péron & Lesueur, 1810) as an exception (Jarms et al. 1999). An additional feature of coronates is the presence of a periderm tube in species that have a known polyp stage (Russell 1970; Jarms 1991).

The high morphological similarity among polyps in this order and the difficulty in relating the polyp to the medusa (as they are usually not collected together) have led to two classification systems. Polyps were historically identified only as “Coronatae polyps” or were classified as belonging to the genus *Stephanoscyphus* Allman, 1874 (later changed to *Stephanoscyphistoma* Jarms, 1990, to accommodate these specimens). Studying the life cycle has been essential for advancing the systematics and understand the evolution of the group (Jarms 1990, 2010). Such an approach allows integration of the two classification systems, ensuring more precise recognition, diagnosis, and eventual identification of species and specimens.

Recent studies have advanced our taxonomic understanding of the group, using morphological characters of the periderm tube to differentiate species relying only on the polyp stage (Morandini and Jarms 2005, 2010, 2012). These characters consist of several tube measurements (total length, opening diameter, and diameter at different heights along the tube, following Jarms 1990, 1991), the number of internal cusps, and differentiation of the tube’s external ornamentation (number of rings). Although useful to distinguish genera and some species, this analysis is not as effective in differentiating all species, especially in the genus *Nausithoe* (Molinari and Morandini 2019). These limitations are even more evident in the sister clade Discomedusae, in which species cannot be identified solely on the polyp stage (van Walraven et al. 2020). Thus, it is important to state that genetic information, together with morphological data, has been fundamental in differentiating and identifying cnidarian species (Dawson 2005; Gómez Daglio and Dawson 2017).

The order Coronatae is composed of six families (13 genera and 59 species), of which Nausithoidae Haeckel, 1880, is the most speciose, with three genera and 26 species (Jarms and Morandini 2019). The most diverse genus, *Nausithoe*, contains 22 accepted species, which are distributed worldwide in shallow to deep waters, and from the tropics to polar regions (Jarms and Morandini 2019). Along the coast and in offshore territorial waters of Brazil, three species of the family have been described or recorded: the medusae of *Nausithoe atlantica* Broch, 1913 and *Nausithoe punctata* Kölliker, 1853, and the polyp of *Nausithoe aurea* Silveira & Morandini, 1997 (Oliveira et al. 2016).

The purpose of this study was to identify coronate polyps from the Brazilian continental slope off Arraial do Cabo (southeastern Brazil, western South Atlantic) and to compare them with the other known species from the Atlantic Ocean and the Brazilian coast, and thus, to improve our knowledge of coronate biodiversity and distribution.

## Materials and methods

**Figure 1.**
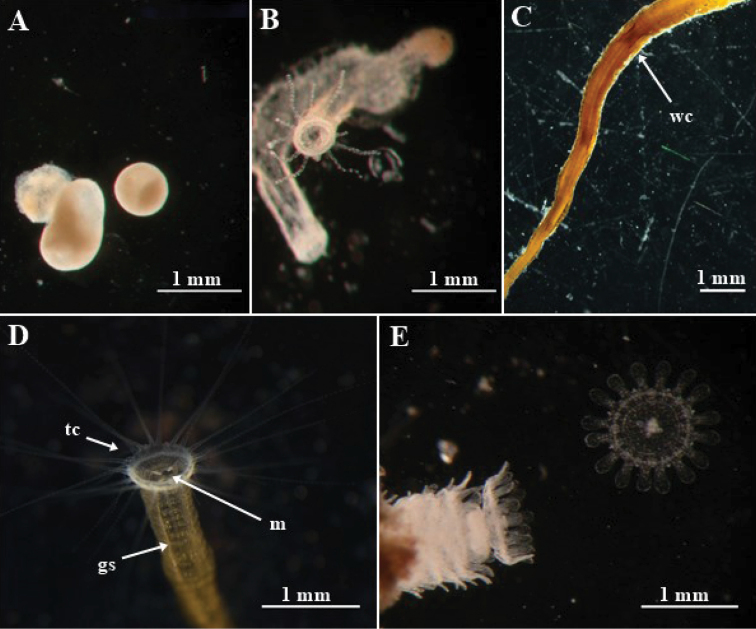
*Nausithoe* sp. from Brazil. **A** tissue balls originated from polyp AC02 ephyrae **B** emerged polyps from same tissue balls in A after 22 weeks, showing the polyp opening; note that the basal parts are fused **C** external view of part of polyp AC18, showing by transparency the internal whorls of cusps (**wc**) **D** oral disc of polyp AC01, showing the tentacle crown (**tc**) and the mouth opening (**m**) with the gastric longitudinal septa (**gs**) **E** polyp AC02 releasing ephyrae.

Deep-sea specimens from Brazil were collected during a cruise of the *Navio Oceanográfico Prof. Wladimir Besnard* off the coast of Arraial do Cabo (Rio de Janeiro state, southeastern Brazil, 23°45.80'S, 41°44.40'W) on 15 September 2002, at a depth of 227 m. Calcareous substrates were collected using a box-corer, and the polyps were found on these. The collected polyps were provisionally named *Nausithoe* sp. and numbered as follows: AC01, AC02, AC08, AC10, AC17, AC18, and AC20 (Table 2). They were kept alive in small dishes for a few days aboard ship (under room temperature 20–23 °C) and brought to the laboratory on 18 September 2002. Since their establishment in culture, three polyps (AC01, AC02, and AC10) reproduced asexually by forming tissue balls from the ephyrae (Fig. 1A, B), consistent with the observations of Silveira et al. (2003).

The study was conducted at the Laboratório de Cultivo e Estudos de Cnidaria, in the Zoology Department of the Biosciences Institute, University of São Paulo (IB–USP). For comparison, we used living polyps of several species: nine *Nausithoe* sp., nine *Nausithoe werneri* Jarms, 1990, 14 *Nausithoe maculata* Jarms, 1990, and three *Nausithoe aurea* (Table 1). They were selected due to their availability in culture at our laboratory and for their distribution in the Atlantic Ocean, which we considered important for comparison.

**Table 1.** Data from studied species of *Nausithose* (*Nausithoe* sp., *N. werneri*, *N. maculata*, and *N. maculata* (= *N. aurea*).

	Locality	# of polyps	Depth (m)	Culture temperature (°C)	Sampling date	Culture codes	# of ephyrae cultivated	# of mature medusae
*Nausithoe* sp.	off Arraial do Cabo, Brazil	9	227	15	15.09.2002	AC01, AC02, AC08, AC10, AC17, AC18, and AC20	850	25
*Nausithoe werneri*	Atlantic off Morocco	4	800–3,000	15	Original culture clones (1980)	ACM156	69	4
*Nausithoe werneri*	Mediterranean	5	200	15	2008	ACM157	70	9
*Nausithoe maculata* (= *N. aurea*)	Ilhabela, Brazil	3	3–9	20–22	02.2018	ACM120	85	0
*Nausithoe maculata*	Girón, Cuba	8	5–10	20–22	02.2004	ACM026, ACM027	50	8
*Nausithoe maculata*	Puerto Rico	6	5–10	20–22	Original culture clones (1971)	ACM025	160	33

Voucher specimens of *Nausithoe* sp. were deposited in the Museu de Zoologia da Universidade de São Paulo as: MZUSP8502 (one medusa), MZUSP8503 (two polyps), MZUSP8504 (50+ ephyrae).

### Cultivation

All polyps were fed with 1–2-day-old nauplii of *Artemia* sp. once a week. Strobilation usually produced hundreds of ephyrae (Fig. 1E), and excessive feeding to increase the number of jellyfish was not necessary. Polyps and ephyrae were kept separately in 200-mL acrylic containers (maximum of 20 ephyrae per dish) in incubation chambers at the appropriate temperature for each species (*N. werneri* and *Nausithoe* sp. at 15 °C; *N. maculata* and *N. aurea* at 20 °C). Once ephyrae were released, they were fed daily with macerated mussel gonads (*Perna perna*). As soon as they grew large enough to catch *Artemia*, we began to vary their diet, adding 1-day-old nauplii. Food was provided in abundance. The water in the polyp dishes was changed 1 day after feeding, and in the medusae and ephyrae containers, it was changed 1 hour after feeding (as these stages are more responsive to variations in water quality). Measurements and photographs of all stages were taken for comparison using a Nikon SMZ800 stereomicroscope and a Nikon Eclipse 80i microscope.

**Table 2.** Measurements of the polyps according to Jarms (1990) and Jarms et al. (2002). Specimens of *Nausithoe* sp. were measured twice, in 2002 and 2018 (except for tissue balls, measured only in 2018). Da = diameter at aperture, Dbd = diameter of basal disc, Db = diameter just above basal disc, D_2 mm_ = diameter at 2 mm height, D_5 mm_ = diameter at 5 mm height, L_tot_ = total length, Nwt = number of whorls of internal cusps, Nw = number of cusps per whorl, – = not measured.

Polyp ID	L_tot_ (mm)	Da (mm)	Ddb (mm)	Db (mm)	D_2 mm_	D_5 mm_	Da/L_tot_	Nwt	Nw
AC01 (2002)	6.60	0.70	–	–	0.35	0.45	0.1061	7	8 / 16
AC01 (2018)	13.46	1.29	–	–	–	–	0.0958		
AC02 (2002)	11.15	0.90	–	0.15	0.30	0.50	0.0807	8	8 / 16
AC02 (2018)	17.74	1.09	–	–	–	–	0.0614		
AC02 (tissue ball 2018)	15.71	1.17	–	0.14	0.09	0.44	0.0746	6	8 / 16
AC08 (2002)	9.10		–	–	0.30	0.50	0.0769	9	8 / 16
AC08 (2018)	16.44	1.00	–	–	–	–	0.0608		
AC10 (2002)	14.25	1.15	–	0.15	0.20	0.40	0.0807	12	8 / 16
AC10 (2018)	15.33	1.15	–	–	–	–	0.0753		
AC10 (tissue ball 2018)	11.79	0.92	0.66	0.15	0.09	0.46	0.0782	6	8 / 16
AC17 (2002)	5.05	0.55	–	0.40	0.45	0.55	0.1089	9	8 / 16
AC17 (2018)	20.20	1.65	–	–	–	–	0.0818		
AC18 (2002)	10.50	0.95	–	0.15	0.35	0.5	0.0905	5	8 / 16
AC18 (2018)	12.27	1.02	–	–	–	–	0.0830		
AC20 (2002)	5.15	0.35	–	0.20	0.30	0.35	0.0680	10	8 / 16
AC20 (2018)	20.13	1.47	–	–	–	–	0.0733		
Mean ± SD (2002)	8.83 ± 3.17	0.77 ± 0.27	–	0.21 ± 0.10	0.32 ± 0.07	0.46 ± 0.06	0.09 ± 0.01	8 ± 3	8 / 16
Mean ± SD (2018)	15.90 ± 2.91	1.20 ± 0.22	0.66 ± 0	0.15 ± 0	0.09 ± 0	0.45 ± 0	0.08 ± 0.01		
*N. werneri* 1	11.35	1.29	0.53	0.11	0.12	0.15	0.1139	6	8
*N. werneri* 2	15.27	0.95	–	0.23	0.11	0.17	0.0620	6	8
*N. werneri* 3	31.43	0.93	0.24	0.07	0.07	0.07	0.0297	7	8
*N. werneri* 4	19.37	1.28	0.40	0.08	0.08	0.11	0.0660	13	8
*N. werneri* 5	13.70	1.02	0.29	0.09	0.09	0.13	0.0744	8	8
*N. werneri* 6	5.24	0.55	–	0.10	0.10	0.11	0.1048	8	8
*N. werneri* 7	13.49	0.68	0.35	0.08	0.09	0.13	0.0502	14	8
*N. werneri* 8	6.47	0.65	0.34	0.08	0.12	0.15	0.1003	8	8
*N. werneri* 9	2.56	0.33	0.30	0.10	–	0.13	0.1293	7	8
Mean ± SD	13.21 ± 8.17	0.85 ± 0.31	0.35 ± 0.09	0.10 ± 0.05	0.10 ± 0.02	0.13 ± 0.03	0.08 ± 0.03	8.56 ± 2.75	8
*N. maculata* 1	13.58	0.88	0.42	0.15	0.12	0.14	0.0646	3	16
*N. maculata* 2	14.41	1.21	–	–	–	–	0.0843	–	16
*N. maculata* 3	15.01	1.18	–	–	–	–	0.0785	–	–
*N. maculata* 4	18.64	1.08	–	–	–	–	0.0578	–	–
*N. maculata* 5	14.60	1.58	–	0.20	0.12	0.19	0.1083	4	16
*N. maculata* 6	15.82	1.29	–	–	–	–	0.0817	–	16
*N. maculata* 7	16.42	0.66	–	0.17	0.09	0.20	0.0405	6	16
*N. maculata* 8	12.85	0.74	0.51	0.10	0.10	0.18	0.0580	–	–
*N. maculata* 9	8.75	0.83	0.41	0.10	0.16	0.19	0.0955	5	16
*N. maculata* 10	3.79	0.39	0.38	0.13	–	0.16	0.1041	4	16
*N. maculata* 11	7.23	0.69	0.30	0.10	0.12	0.19	0.0949	4	16
*N. maculata* 12	12.72	0.64	0.41	0.13	0.12	0.16	0.0504	8	16
*N. maculata* 13	4.91	0.77	–	–	0.14	0.21	0.1576	5	16
*N. maculata* 14	8.01	1.19	–	0.11	0.11	0.17	0.1382	3	16
Mean ± SD	11.91 ± 4.39	0.94 ± 0.06	0.41 ± 0.06	0.13 ± 0.03	0.12 ± 0.02	0.18 ± 0.02	0.09 ± 0.03	4.67 ± 1.49	16
*N. maculata* (= *N. aurea*) 1	3.93	0.38	0.17	0.08	–	0.17	0.0979	6	16
*N. maculata* (= *N. aurea*) 2	3.86	0.53	0.36	0.11	–	0.16	0.1388	5	16
*N. maculata* (= *N. aurea*) 3	5.47	0.60	0.60	0.13	0.11	0.19	0.1091	6	16
Mean ± SD	4.42 ± 0.74	0.50 ± 0.09	0.38 ± 0.18	0.11 ± 0.02	0.11 ± 0	0.17 ± 0.01	0.12 ± 0.02	5.67 ± 0.47	16

### Morphological analyses

**Figure 2.**
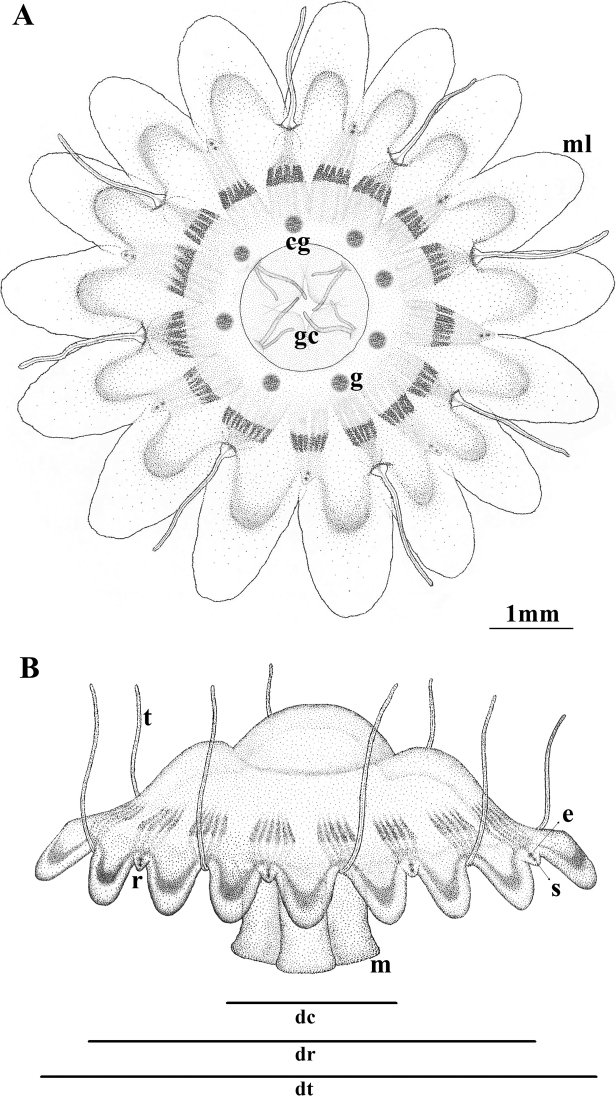
Schematic view of a typical *Nausithoe* sp. adult medusa, illustrating the main characters. **A** aboral view **B** lateral view. **dc** diameter of coronal furrow **dr** diameter between rhopalia **dt** total diameter **cg** coronal grove **gc** gastric cirri **m** manubrium **ml** marginal lappets **r** rhopalium **t** tentacle **e** eyespot **g** gonad **s** statolith.

Measurements followed the protocols established by Jarms (1990, 1991) and Jarms et al. (2002). The main measurements were: i) total tube length, ii) tube diameter at 2 mm above the base, iii) tube diameter at 5 mm above the base, iv) diameter of the basal disc, v) diameter just above the basal disc, and vi) aperture diameter. The type of external ornamentation (number of transverse rings per 4-mm length) and whether these rings were more or less prominent with respect to an imaginary line tangential to the tube contour was noted for possible comparison (according to Morandini and Jarms 2005). For the more translucent tubes, we were able to observe the number of whorls of the cusps and the number of cusps per whorl. We also took scanning electron microscope (SEM) photographs of the internal cusps of some polyps (*Nausithoe* sp. and *N. werneri*) in order to observe their shape and ornamentation (with special attention to whether they had additional cusps on the margin and surface). Characters and measurements of the ephyrae and medusae are shown in Fig. 2. The SEM observations were conducted at the Laboratório de Biologia Celular e Microscopia of the IB–USP, and the method of preparing the specimens followed the protocols of Morandini and Jarms (2005, 2010, 2012).

### Cnidome analyses

Capsule types and sizes of nematocysts in the different life-cycle stages (polyp, ephyra, medusa) were measured (Mariscal 1974; Östman 2000) using a Nikon Eclipse 80i light microscope. A total of 60 measurements were performed on each type per life-cycle stage of *Nausithoe* sp. and on the medusa tentacles of *N. werneri* and *N. maculata*.

### DNA analyses

Because hundreds of ephyrae were produced, most of the molecular tissue for DNA extractions came from them (and some from mature medusae). We extracted DNA from specimens representing all the putative species in this study: *Nausithoe* sp., *Nausithoe aurea*, *Nausithoe maculata*, and *Nausithoe werneri*. DNA was extracted using an ammonium acetate-based protocol adapted from Fetzner (1999). Preliminary tests established that the minimum number of ephyrae for a satisfactory extraction was around 30, considering their size. Three partial genes were amplified by polymerase chain reaction (PCR): mitochondrial protein coding cytochrome oxidase subunit I (COI), and nuclear ribosomal markers 18S and 28S, using published primers and PCR conditions (Table 3). PCR products were purified with the Agencourt AMPure kit (#A63881). The BigDye reaction was conducted using the same primers and annealing temperature in each case. These steps were performed in the Laboratório de Evolução Molecular of the Zoology Department (IB–USP). Finally, the precipitated DNA were sequenced at the Laboratório de Sinalização de Redes Regulatórias de Plantas at the Botanical Department, IB–USP, with a Hitachi 3730xl DNA Analyzer. Chromatograms (.abi files) and consensus sequences were created and checked to identify potential sequencing errors and/or contamination, using Geneious software 9.1 (Kearse et al. 2012; all COI sequences were translated to check potential mitochondrial pseudogenes (NUMTs) or indel artifacts – genetic code: Invertebrate Mitochondrial). Sequence alignments were performed with the Geneious 9.1 MAFFT module for 18S and 28S markers (auto-mode; Katoh and Standley 2013), and Translation Align module for COI marker (genetic code: Invertebrate Mitochondrial; MAFFT alignment: E-INS-i); this way, we avoid inserting any spurious gap in the COI alignment. To avoid any difference (artifact) on COI distances (the most relevant dataset to discuss species delimitation), we edited ends of COI alignment. Doing so, all sequences present the same basic information (sequence length). Corrected distance values from each molecular marker alignment were obtained using MEGA X – Kimura 2 Parameters (Kumar et al. 2018).

**Table 3.** List of primers used to amplify each gene. *LEM = Laboratório de Evolução Molecular (IB–USP).

Gene	Primer	Primer sequence 5'–3'	F/R	Temp. – base pairs	Source
COI	COXl-F2	TCGACTAATCATAAAGATATCGGCAC	F	52 °C – 26 bp	Ward et al. 2005
	MEDCOXR	TGGTGNGCYCANACNATRAANCC	R	52 °C – 23 bp	Lawley et al. 2016
	LCO1490JJ4	CIACIAAYCAYAARGAYATYGG	F	55 °C+45 °C – 22 bp	Astrin et al. 2016
	HCO2198JJ4	ANACTTCNGGRTGNCCAAARAATC	R	55 °C+45 °C – 25 bp	Astrin et al. 2016
	LCO1490JJ2	CHACWAAYCAYAARGAYATYGG	F	60 °C+45 °C – 22 bp	Astrin et al. 2016
	HCO2198JJ2	ANACTTCNGGRTGNCCAAARAATCA	R	60 °C+45 °C – 25 bp	Astrin et al. 2016
28S	F15	CTAACAAGGATTCCCCTAGTAACGGCGAG	F	55.5 °C – 30 bp	LEM *
	R798	GGTCCGTGTTTCAAGACGG	R	55.5 °C – 19 bp	Medina et al. 2001
	F798	CCGTCTTGAAACACGGACC	F	55.5 °C – 19 bp	Medina et al. 2001
	R1446	GTTGTTACACACTCCTTAGCGG	R	55.5 °C – 22 bp	Medina et al. 2001
18S	18S – A	AACCTGGTTGATCCTGCCAGT	F	54 °C – 21 bp	Medlin et al. 1988
	18S – L	CCAACTACGAGCTTTTTAACTG	R	54 °C – 22 bp	Apakupakul et al. 1999
	18S – C	CGGTAATTCCAGCTCCAATAG	F	54 °C – 21 bp	Apakupakul et al. 1999
	18S – Y	CAGACAAATCGCTCCACCAAC	R	54 °C – 21 bp	Apakupakul et al. 1999
	18S – O	AAGGGCACCACCAGGAGTGGAG	F	54 °C – 22 bp	Apakupakul et al. 1999
	18S – B	TGATCCTTCCGCAGGTTCACCT	R	54 °C – 22 bp	Medlin et al. 1988

## Results

All the morphological features observed on the available specimens are summarized in Tables 2, 4. The time periods until medusae developed distinct morphological characters are listed in Table 5.

**Table 4.** Morphological data for studied species of *Nausithoe*. Data combined from literature and observations on specimens. Brazilian state abbreviations: BA, Bahia; RJ, Rio de Janeiro; SP, São Paulo.

		***N. sp.***	***N. maculata* (= *N. aurea*)**	***N. maculata***	***N. werneri***
Medusae	Number / shape of lappets	16, slightly elongate with rounded margins	16, slightly elongate with rounded margins	16, slightly elongate with rounded margins	16, slightly elongate with rounded margins
	Rhopalium with ocellus	yes	yes	yes	yes
	Number of gastric filaments	8 (2 × 4)	12–24 (3–6 × 4)	28 (7 × 4)	4–12 (1–3 × 4)
	Gonad shape	round	round	round	round
	Central disc shape	central dome slightly elevated	flattened	flattened	central dome elevated
	Total diameter (mm) / Central disc diameter (mm)	9.5 / 2.83	5 / 1.9	4 / 1.5	12 / 4.5
	General coloration	transparent	transparent with yellow pigment spot on lappets	transparent with yellow pigment spot on lappets	transparent
	Gonad color	dark brown	yellow to brown	yellow to brown	dark brown
	Tentacle length (mm)	5	1.5	2	3
Polyp	Habitus	solitary	solitary	solitary	solitary
	Occurrence	off Cabo Frio, RJ (Brazil)	Brazil (SP to BA)	Puerto Rico; Cuba	Atlantic off Morocco; Greenland
	Depth (m)	227	3–9	5–10	200–3000
	Length (mm)	5.05–20.2	1.35–9.18	3.79–18.64	2.56–31.46
	Number of cusps/whorls	8 + 16	16	16	8
	Number of whorls of cusps	3–10	2–7	3–8	6–14

**Table 5.** Development times (in weeks, under laboratory conditions) of several structures in the species studied.

	*N. werneri*	*N. maculata* (= *N. aurea*)	*N. maculata*	*N. sp.*
Ocelli	4	2	3	3
First gastric filament	3	1	2	2
Second gastric filament	–	–	8	13
Tentacle buds	7	3	3	2
Lappets, pigment spot	–	2	2	–
Gonads	9	3	4	24

### Systematics section

**Class Scyphozoa Goette, 1887**

**Subclass Coronamedusae Calder, 2009**

**Order Coronatae Vanhöffen, 1892**

**Family Nausithoidae Haeckel, 1880**

#### Genus *Nausithoe* Kölliker, 1853

##### Nausithoe

Taxon classificationAnimaliaCoronataeNausithoidae

sp.

623C3844-D8A5-5D83-A52E-8C0B5B460D5D

Figs 1 , 3 , 4

###### Metagenetic species.

**Figure 3.**
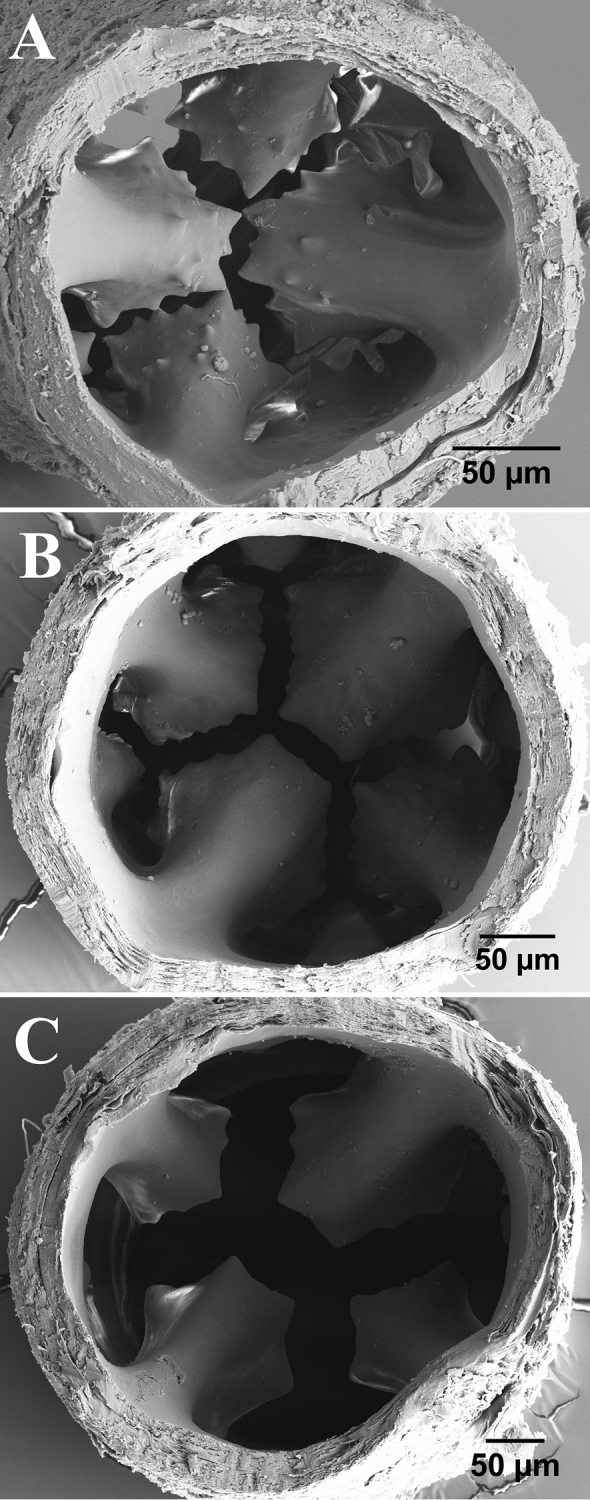
SEM of cross-sections of the tube of the polyp of *Nausithoe* sp. AC02 at different heights. **A** more-basal series, with 16 cusps and additional cusps over 4 larger perradial cusps **B** and **C** two more-distal series (**C** being the highest along the tube), each with 8 cusps.

**Figure 4.**
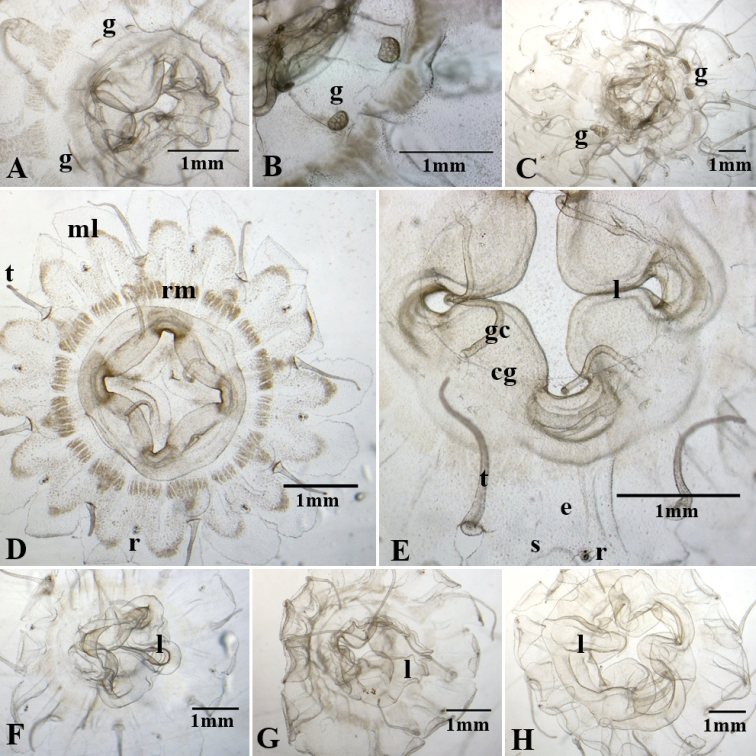
Adult medusae from polyps of *Nausithoe* sp. AC10 (**A–C**) and AC20 (**D–H**) **A** beginning of gonad (**g**) development **B** two gonads with gametic cells still differentiating **C** general view of a medusa that we managed to maintain until the gonads emerged (note degree of irregularities in this specimen, due to the long period in cultivation) **D** aboral view of 3-month-old medusa, showing the radial muscle (**rm**), marginal lappets (**ml**), rhopalium (**r**), and tentacles (**t**) **E** detail of 6-month-old medusa, showing gastric filaments (**gf**), rhopalium with ocelli (**e**), and statocyst (**s**), and coronal groove (**cg**); note, no trace of gonad development **F**, **G** and **H** Oral view of medusae, showing lips (**l**) and projection of the manubrium, from less projected (**F**) to more projected (**H**).

**Figure 5.**
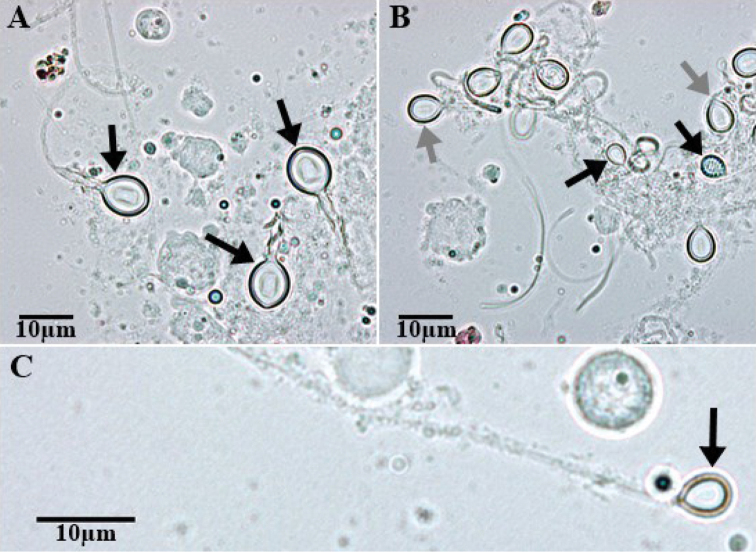
Photomicrographs of the two nematocyst types found in *Nausithoe* sp. **A** three heterotrichous microbasic euryteles discharged **B** holotrichous isorhiza capsules of two different sizes (large, grey arrow; small, black arrow) **C** a small discharged holotrichous isorhiza.

Solitary polyp with typical periderm tube, dark to light brown (Fig. 1C, D), conical, with transverse rings on the surface with longitudinal striations (5 rings every 0.4 mm at 2 mm above base). Polyps 5.05–20.2 mm long; basal disk 0.63–0.66 mm in diameter; diameter just above the basal disk 0.14–0.4 mm; tube diameter at 2-mm height 0.09–0.45 mm, and at 5-mm height 0.35–0.45 mm; tube aperture diameter 1.28 mm. Tubes with 3–10 whorls of internal cusps (Fig. 1C); closer to base, number of internal cusps per whorl is 16: 4 large (perradius) with additional cusps on the surface, 4 intermediate (interradius), and 8 small (adradius) (Fig. 3A). Upper whorls with only 8 cusps: 4 large (perradius) with no additional cusps, and 4 intermediate (interradius) (Fig. 3B, C). Polyps with 26–37 filiform tentacles (Fig. 1D). Polydisc strobilation, with more than 100 ephyrae at a time. Medusa (Figs 2A, B, 4) entirely translucent, with slightly flattened smooth umbrella; 16 slightly elongated lappets with rounded margins; 8 rhopalia with statocyst and red ocelli. Live specimens measuring up to: 9.5 mm total diameter; 7.74 mm diameter between opposite rhopalia; 2.83 mm coronal groove diameter; gastric cirri approximately 0.9 mm in length; and tentacle length up to half the total diameter of the medusa. Stomach with 4 gastric septa, each with 2 gastric filaments (8 in total) (Figs 2A, 4D, E). Gonads, 8, but not fully developed (Fig. 4A–C); in fact, only a single individual had them and they were malformed (Fig. 4B, C), so complete data about their morphology are lacking. Cnidome composed of only two nematocyst types: holotrichous isorhiza and heterotrichous microbasic eurytele (Fig. 5; Table 6).

**Table 6.** Cnidome of studied species of *Nausithoe*. The range was obtained from 60 nematocysts of each type at each stage. *Data from Silveira and Morandini (1997; from 20 nematocysts of each type at each stage).

		Holotrichous isorhiza		Heterotrichous microbasic eurytele	
		Width (µm)	Length (µm)	Width (µm)	Length (µm)
*Nausithoe* sp.	medusa (tentacle)	4.41–6.49	5.65–8.65	7.28–10.96	8.47–12.99
	ephyra (whole)	4.11–7.33	5.29–8.98	7.1–12.59	8.19–14.27
	polyp (tentacle)	5.86–7.58	8.24–9.67	9.58–11.54	11.28–13.53
	medusa (tentacle)	3.0–4.2	4.2–6.0	5.4–7.2	6.6–9.0
*N. maculata* (= *N. aurea*)*	ephyra (whole)	3.6–6.0	5.4–7.2	9.0–11.4	10.2–12.6
	polyp (tentacle)	3.6–5.4	6.0–7.8	3.0–4.8	9.0–15.0
*N. maculata*	medusa (tentacle)	3.52–5.08	4.5–5.83	6.49–8.17	7.49–9.21
*N. werneri*	medusa (tentacle)	4.33–6.69	5.47–7.45	7.06–9.27	8.67–10.92

##### Nausithoe maculata

Taxon classificationAnimaliaCoronataeNausithoidae

Jarms, 1990

558AC9DD-EC38-53AF-B23F-1E284E63406E

Fig. 6

Nausithoe maculata Jarms 1990: 21–24, figs 15–17, pl. V. Type locality: Puerto Rico. Holotype: ZMH C11534.
Nausithoe aurea Silveira and Morandini 1997: 236–239, figs 1–7, pls I, II. Type locality: Ilhabela (23°51'S, 45°25'W), Brazil. Holotype: MNRJ 2899. (syn. nov.)

###### Metagenetic species.

**Figure 6.**
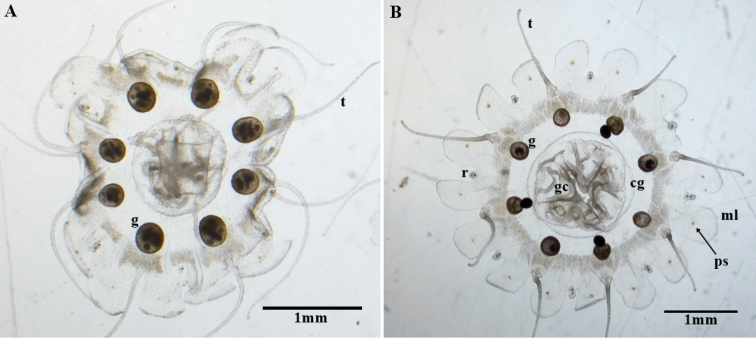
Aboral view of *Nausithoe maculata* medusae from Cuba’s polyp culture. **A** four-month-old medusa with mature male gonads (**g**), long tentacles (**t**), and malformations in the lappets and central disc. **B** three-month-old medusa with mature female gonads (**g**), showing the rhopalium (**r**), coronal groove (**cg**), marginal lappets (**ml**) with pigment spot (**ps**), gastric cirri (**gc**), and tentacles (**t**).

Solitary polyp with typical periderm tube, dark to light brown, conical, with transverse rings on the surface with longitudinal striations (3–5 rings every 0.4 mm at 2 mm above base). Polyps 3.79–18.64 mm long. Basal disk 0.17–0.6 mm in diameter. Diameter of aperture 0.39–1.58 mm. Diameter just above the basal disc 0.08–0.2 mm. Diameter at 2-mm height 0.09–0.16 mm, and at 5-mm height 0.14–0.21 mm. Three to 8 whorls of 16 internal smooth cusps: 4 large (perradius), 4 intermediate (interradius), and 8 small (adradius). Polydisc strobilation, with more than 100 ephyrae at a time. Medusa translucent, with yellow pigment spot in center of each lappet (Fig. 6A, B). Umbrella flattened, with 16 slightly elongated lappets with rounded margins and 8 rhopalia with statocyst and red ocelli. Live specimens measuring up to: 3.9 mm total diameter; 2.7 mm between opposite rhopalia; 1.5 mm coronal-groove diameter; gastric cirri approximately 0.5 mm in length; and tentacle length up to 1.5 mm. Stomach with 4 gastric septa, each with 3–7 gastric filaments.

##### Nausithoe werneri

Taxon classificationAnimaliaCoronataeNausithoidae

Jarms, 1990

0F6367D3-3578-5575-BABB-F5919BDF0758

Fig. 7

Nausithoe werneri Jarms 1990: 12–17, figs 1–7, pls I, III. Type locality: Morocco coast (25°20.4'N, 16°08.4'W, 415–420 m depth). Holotype: ZMH C11530.

###### Metagenetic species.

**Figure 7.**
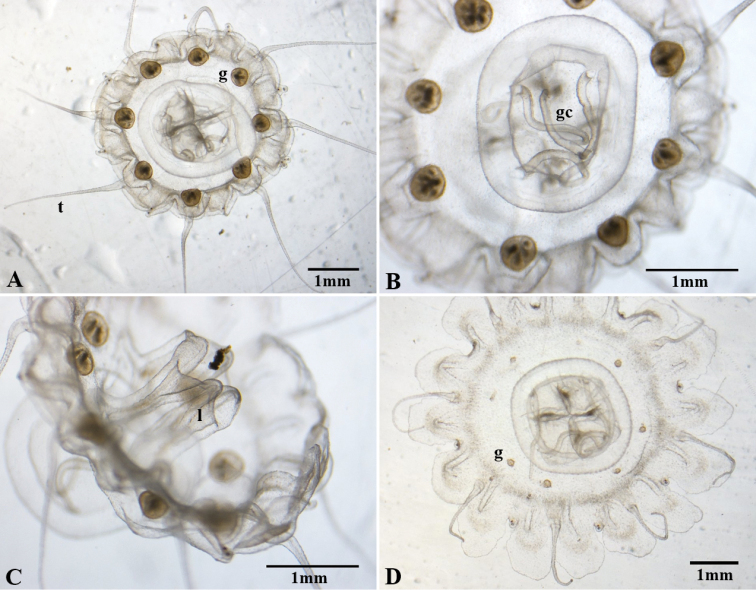
*Nausithoe werneri* male medusae from Mediterranean’s polyp culture (**A**–**C** 5 months old **D** 3 months old) **A** aboral view of an adult medusa with mature gonads (**g**), contracted lappets, and extended tentacles (**t**) **B** detail of gastrovascular cavity, focusing on the gastric cirri (**gc**) **C** lateral view, focusing on the lips (**l**) and the extension of the manubrium **D** beginning of gonad (**g**) development (aboral view).

Solitary polyp with typical periderm tube, dark to light brown, conical, with transverse rings on surface with longitudinal striations (4–5 rings every 0.4 mm at 2 mm above the base). Polyps 2.56–31.46 mm long. Basal disk 0.24–0.53 mm in diameter. Diameter of aperture 0.33–1.29 mm. Diameter just above basal disc 0.07–0.23 mm. Diameter at 2-mm height 0.07–0.12 mm, and at 5-mm height 0.07–0.17 mm. Six to 14 whorls of 8 internal cusps: 4 large (perradius) and 4 intermediate (interradius), with additional cusps. Polyp with up to 40 filiform tentacles. Polydisc strobilation, with more than 100 ephyrae at a time. Medusa with smooth umbrella (Fig. 7) with high central dome; 16 slightly elongated lappets with rounded margins, and 8 rhopalia with statocyst and red ocelli. Live specimens measuring up to: 6.8 mm total diameter; 5 mm between opposite rhopalia; 2.5 mm coronal groove diameter; gastric cirri approximately 1.9 mm in length; and tentacle length up to 3 mm. Stomach with 4 gastric septa, each with 1 gastric filament (4 in total).

Sequences from all species studied are available on GenBank (Table 7). DNA comparisons of the sequenced markers of the species are summarized in Tables 8 and 9. We were not able to amplify the three proposed markers for all polyps. Therefore, *Nausithoe* sp. specimens AC01 (no 28S data), AC17 (no COI data), and AC18 (no COI data) were not included in the molecular analyses. Polyps with all three genes sequenced were compared with each other and with *N. maculata* (= *N. aurea* from Brazil), *N. maculata* (Cuba), and *N. werneri*. Using only the COI marker, *Nausithoe* sp. (specimens AC02, AC08, AC10, AC20) and *N. werneri* had less than 6% genetic difference from each other and almost 20% genetic difference from *N. maculata* (Cuba) and *N. maculata* (= *N. aurea* from Brazil). *Nausithoe maculata* from Cuba had less than 7% difference from *N. maculata* (= *N. aurea*) from Brazil. The remaining DNA extractions from cultures are also considered as vouchers (Table 7).

**Table 7.** Sequence accession numbers in GenBank and their respective DNA extraction vouchers.

	COI	18S	28S	DNA Voucher
*Nausithoe* sp. AC02	MT603856 (717 bp)	MT603629 (1765 bp)	MT621557 (1304 bp)	N02
*Nausithoe* sp. AC08	MT603855 (735 bp)	MT603631 (1767 bp)	MT621552 (1309 bp)	N08
*Nausithoe* sp. AC10	MT603857 (708 bp)	MT603630 (1765 bp)	MT621553 (1319 bp)	N10
*Nausithoe* sp. AC20	MT603854 (609 bp)	MT603628 (1772 bp)	MT621555 (1344 bp)	N20
*N. werneri* (Mediterranean)	MT603858 (610 bp)	MT603627 (1767 bp)	MT621554 (1337 bp)	NW (Med)
*N. maculata* (= *N. aurea*) (Brazil)	MT603859 (579 bp)	MT603632 (1777 bp)	MT621558 (1305 bp)	NA
*N. maculata* (Cuba)	MT603860 (591 bp)	MT603633 (1780 bp)	MT621559 (1310 bp)	NM (Cuba)

**Table 8.** Genetic similarity (percent) between each *Nausithoe* sp. polyp (AC02, AC08, AC10, AC20), *Nausithoe maculata* (= *N. aurea* from Brazil), *Nausithoe maculata* (from Cuba), and *Nausithoe werneri*. 18S in white and 28S in italic.

	AC02	AC08	AC10	AC20	*N. werneri*	*N. maculata* (Brazil)	*N. maculata* (Cuba)
AC02		100	100	99.94	99.94	99.86	99.86
AC08	*94.86*		100	99.94	99.94	99.86	99.86
AC10	*97.04*	*94.63*		99.94	99.94	99.86	99.86
AC20	*97.41*	*95*	*97.18*		99.89	99.80	99.80
*N. werneri*	*97.22*	*94.69*	*96.91*	*97.28*		99.80	99.80
*N. maculata* (Brazil)	*96.64*	*94.22*	*96.62*	*96.76*	*96.51*		99.94
*N. maculata* (Cuba)	*96.66*	*94.18*	*96.37*	*96.74*	*96.47*	*97.36*	

**Table 9.** Genetic similarity (percent) between each *Nausithoe* sp. polyp (AC02, AC08, AC10, AC20), *N. maculata* (= *N. aurea* from Brazil), *N. maculata* (from Cuba), and *N. werneri*. COI in italic; all three markers combined in white. Boldface indicates higher similarity that we are considering to be the same species.

	AC02	AC08	AC10	AC20	*N. werneri*	*N. maculata* (Brazil)	*N. maculata* (Cuba)
AC02		96.28	97.33	94.60	95.35	91.74	92.08
AC08	***97.35***		96.11	94.1	94.28	90.99	91.39
AC10	***100***	***97.32***		94.73	95.32	91.77	92.11
AC20	***94.25***	***95.40***	***94.25***		94.73	92.00	92.18
*N. werneri*	***100***	***97.21***	***100***	***94.06***		91.72	92.05
*N. maculata* Brazil)	*80.83*	*80.83*	*80.83*	*81.35*	*80.83*		94.06
*N. maculata* (Cuba)	*81.89*	*82.40*	*81.89*	*82.29*	*81.89*	***93.06***	

## Discussion

The objective of this study was to identify the polyps of *Nausithoe* sp. from deep waters off southeastern Brazil by comparing them with previous records along the Brazilian coast. We used two approaches: morphology combined with life-cycle observations, and molecular data. Also, we present previously unpublished data on nematocysts for *N. werneri* and *N. maculata* from Cuba.

So far, only three species of *Nausithoe* have been recorded from the Brazilian coast: *N. aurea*, *N. atlantica*, and *N. punctata* (Oliveira et al. 2016). *Nausithoe aurea* is endemic to the Brazilian coast and can be found in shallow waters (Silveira and Morandini 1997; Morandini and Silveira 2001). The polyps are solitary with 16 smooth cusps per series; and the medusae have a characteristic single yellow spot in the center of each marginal lappet (Silveira and Morandini 1997). These features show that this species is not identical to *Nausithoe* sp.

*Nausithoe punctata* is a cosmopolitan species that lives in shallow waters (Jarms and Morandini 2019); in Brazil it was reported by Goy (1979) and Neumann-Leitão et al. (2008). The polyp stage is colonial and inhabits sponges (Werner 1979; Uriz et al. 1992). Medusae are transparent with a pale-pink disc and yellowish lappets; the umbrella is flattened and reaches up to 15 mm in diameter (Kölliker 1853; Kramp 1961; Werner 1970). With these features, we can also discard this species as identical to *Nausithoe* sp. As pointed out by Jarms and Morandini (2019), there is extensive confusion in the taxonomy and identification of *N. punctata*, and many records around the world might be erroneous.

*Nausithoe atlantica* is known only from the medusa stage (Jarms and Morandini 2019). Although records from the Pacific Ocean exist (Oliveira et al. 2016), the only confirmed identifications are from the North Atlantic, near the type locality (Broch 1913; Russell 1956). The adult medusa is dark yellowish-brown, up to 35 mm in diameter, and has rhopalia without ocelli, oblong gonads, and more than 160 gastric filaments (Russell 1970). The Brazilian record provided by Oliveira et al. (2016) derives from a thesis in which the specimens were collected off Santa Catarina state. The description provided is identical to Russell (1970), and no voucher material is available for comparison. Considering the divergent features and doubtful record, we discard this species as identical to *Nausithoe* sp.

The two types of nematocysts found in *Nausithoe* sp. (heterotrichous microbasic euryteles and holotrichous isorhizas) are the same as in *N. aurea* (Silveira and Morandini 1997) and in *N. planulophora* (Werner 1971). These are the most common types in scyphozoans (Östman 2000), which indicate that this information might not be useful in differentiating species of the group. There is an overlap of the measurements obtained from *Nausithoe* sp. and *N. werneri*, and polyp nematocysts are slightly larger than ephyrae and medusae ones. For now, much more work on the scyphozoan cnidome is needed to improve understanding of the types of cnidae in the group’s evolutionary history.

As do most of the solitary Nausithoidae polyps, those of *Nausithoe* sp. and *N. werneri* resemble each other. As stated by several authors, the most useful features to distinguish *Nausithoe* polyps are the number and shape of the internal cusps of the tube (e.g., Jarms 1991; Morandini and Jarms 2012). Although these features are used widely in the systematics of the group, the variation among specimens has not been thoroughly studied, in part because of the relatively few samples that can be used for scanning electron microscopy. It is also unknown how laboratory conditions might affect the growth of the animals and the shape of the cusps. SEM observations of the cusps of *Nausithoe* sp. showed 16 internal cusps per whorl close to the base and eight cusps in the upper whorls of the tube (Fig. 3), whereas in *N. werneri*, only eight cusps are found per whorl, both in the literature (Jarms 1990) and in our observations.

Both the ephyrae and medusae of the Brazilian deep-sea *Nausithoe* sp. are morphologically similar to *N. werneri*: translucent body, rhopalium with statocyst and red ocelli, lappets slightly elongated with rounded margins, and total diameter (Table 4). The only differences we noted were the shape and size of the manubrium, the shape of the central disc, and the number of gastric cirri. In *Nausithoe* sp., the manubrium is wider (Fig. 4F–H) than in *N. werneri* (Fig. 6D). The elevated central dome of *N. werneri* is also not present in *Nausithoe* sp. Although Jarms (1990) described *N. werneri* with two or three gastric cirri per quadrant, our cultivated specimens (derived from Jarms’s culture) had only one gastric cirrus per quadrant (Fig. 6B); *Nausithoe* sp. has eight filaments in the stomach. These variations in morphological features might be related to plasticity in the response of the species under different environmental conditions and food supply. Certainly, the food provided in our laboratory differs from that used by Jarms (1990). Although our specimens were kept in the same conditions, there might be some individual variation. To ascertain the utility of these morphological features it would be necessary to examine many specimens from a broad population.

An interesting feature is the difference in the time taken to develop gonads between *Nausithoe* sp. and our specimens of *N. werneri* (Table 5). *Nausithoe* sp. took more than 20 weeks to begin the differentiation and development of the reproductive organs, while *N. werneri* required only nine weeks. These times are comparable because all species were kept in the same kind of container and temperature, with similar population densities, and fed equally.

Genetic divergence related to species delimitation is difficult to discern, especially for clades with limited molecular markers and specimens. For Discomedusae, the sister clade of Coronamedusae, Gómez Daglio and Dawson (2017) proposed that the mean intraspecific pairwise genetic distance (x ± s.d.) is 0.006 ± 0.005 and the mean interspecific distance between congeneric species (x ± s.d.) is 0.12 ± 0.04. Our molecular data showed that specimens of *Nausithoe* sp. and *N. werneri* had less than 6% of genetic difference for the COI gene (Table 8) and each of them differed by ~20% from *N. maculata* (from Cuba) and *N. maculata* (= *N. aurea* from Brazil). Together with the morphological similarities, we state they are the same species, according to the criteria of Gómez Daglio and Dawson (2017). Following the same rationale, we found less than 7% of genetic difference in the COI gene between *N. maculata* (from Cuba) and *N. maculata* (= *N. aurea* from Brazil). In these two species, the morphological resemblance is obvious, and the only difference is the formation of planuloids inside the periderm tube of *N. aurea* (Silveira and Morandini 1997), which was considered one of the distinguishing features of this species, not observed in *N. maculata*. Considering that coronamedusae species were mostly defined by a few specimens, we propose that this feature represents possible genetic plasticity not previously recorded for *N. maculata*.

Comparing the life cycle and morphology of scyphomedusae is extremely important to help in identifying and describing species. However, the simple structure of these animals, as evidenced by the traditional use of certain uninformative characters in the description of specimens (e.g., Kramp 1961; Russell 1970), can yield insufficient information for a precise analysis (Dawson 2005). Future approaches, including additional genetic data from other species of Coronatae, will add detail to the systematics of this clade. A broader sampling of molecular markers, individuals, populations, species, and clades in general will allow for novel insights to be applied to Coronamedusae, the proposal of a more refined “genetic gap” for the delimitation of species, and specific genetic diagnoses (DeSalle and Goldstein 2019). Nevertheless, the use of genetic data must be combined with information on morphology and life cycles to confirm or reject the validity of species and to identify new taxa.

To conclude, based on both the morphological and molecular data obtained, we identify the deep-sea *Nausithoe* sp. specimens from off the Brazilian coast as *Nausithoe werneri*, thus expanding the distribution of this species to the western South Atlantic. Additionally and also based on molecular and morphological data, we consider the species *Nausithoe aurea* as a junior synonym of *Nausithoe maculata*.

## Supplementary Material

XML Treatment for Nausithoe

XML Treatment for Nausithoe maculata

XML Treatment for Nausithoe werneri

## References

[B1] Allman GJ (1874) Report on the Hydroida collected during the Expeditions of H.M.S. ‘Porcupine’. Transactions of the Zoological Society of London 8(8): 469–481. [pls 65–68] 10.1111/j.1096-3642.1874.tb00566.x

[B2] Broch H (1913) Scyphomedusae from the “Michael Sars” North Atlantic Deep-Sea Expedition 1910. Report on the Scientific Results of the “Michael Sars” North Atlantic Deep-Sea Expedition 3(4): 1–20. [pl. I]

[B3] Calder DR (2009) Cubozoan and scyphozoan jellyfishes of the Carolinian biogeographic province, southeastern USA. Royal Ontario Museum Contributions in Science 3: 1–58.

[B4] Collins AG (2009) Recent insights into cnidarian phylogeny. Smithsonian Contributions to the Marine Sciences 38: 139–149.

[B5] Dawson MN (2005) Renaissance taxonomy: integrative evolutionary analyses in the classification of Scyphozoa. Journal of the Marine Biological Association of the UK 85(3): 733–739. 10.1017/S0025315405011641

[B6] Dawson MN Jacobs DK (2001) Molecular evidence for cryptic species of *Aurelia aurita* (Cnidaria, Scyphozoa). The Biological Bulletin 200(1): 92–96. 10.2307/1543089 11249217

[B7] DeSalle R Paul Goldstein P (2019) Review and interpretation of trends in DNA barcoding. Frontiers in Ecology and Evolution 7: 1–11. 10.3389/fevo.2019.00302

[B8] Eschscholtz F (1829) System der Acalephen. Eine ausführliche Beschreibung aller Medusenartigen Strahlthiere. Ferdinand Dümmler, Berlin, 190 pp [16 plates] 10.5962/bhl.title.10139

[B9] Fetzner JW (1999) Extracting high-quality DNA from shed reptile skins: a simplified method. Biotechniques 26(6): 1052–1054. 10.2144/99266bm09 10376138

[B10] Goette A (1886) Verzeichnis der Medusen, welche von Dr Sander, Stabsarzt auf S.M.S. “Prinz Adalbert” gesammelt wurden. Sitzungsberichte der Königlich Preussischen Akademie der Wissenschaften zu Berlin 39: 831–837.

[B11] Gómez Daglio L Dawson MN (2017) Species richness of jellyfishes (Scyphozoa: Discomedusae) in the Tropical Eastern Pacific: missed taxa, molecules, and morphology match in a biodiversity hotspot. Invertebrate Systematics 31: 635–663. 10.1071/IS16055

[B12] Goy J (1979) Campagne de la Calypso au large des côtes atlantiques de l’Amérique du Sud (1961–1962) – 35. Méduses. Annales de l’Institut Océanographique, Paris 55 (Suppl.): 263–296.

[B13] Haeckel E (1880) Das System der Medusen. I, 2: System der Acraspeden. Gustav Fischer, Jena, 361–672.

[B14] Holland BS Dawson MN Crow GL Hofmann DK (2004) Global phylogeography of *Cassiopea* (Scyphozoa: Rhizostomeae): molecular evidence for cryptic species and multiple invasions of the Hawaiian Islands. Marine Biology 145: 1119–1128. 10.1007/s00227-004-1409-4

[B15] Holst S Laakmann S (2014) Morphological and molecular discrimination of two closely related jellyfish species, *Cyanea capillata* and *C. lamarckii* (Cnidaria, Scyphozoa), from the northeast Atlantic. Journal of Plankton Research 36(1): 48–63. 10.1093/plankt/fbt093

[B16] Jarms G (1990) Neubeschreibung dreier Arten der Gattung *Nausithoe* (Coronatae, Scyphozoa) sowie Wiederbeschreibung der Art *Nausithoe marginata* Kölliker, 1853. Mitteilungen aus dem Hamburgischen Zoologischen Museum und Institut 87: 7–39.

[B17] Jarms G (1991) Taxonomic characters from the polyp tubes of coronate medusae (Scyphozoa, Coronatae). Hydrobiologia 216: 463–470. 10.1007/BF00026500

[B18] Jarms G (2010) The early life history of Scyphozoa with emphasis on Coronatae. A review with a list of described life cycles. Verhandlungen des Naturwissenschaftlichen Vereins in Hamburg 45: 17–31.

[B19] Jarms G Morandini AC (2019) Coronamedusae In: Jarms G Morandini AC (Eds) World Atlas of Jellyfish.Abhandlungen des Naturwissenschaftlichen Vereins, Hamburg, 112–259.

[B20] Jarms G Båmstedt U Tiemann H Martinussen MB Fosså JH (1999) The holopelagic life cycle of the deep-sea medusa *Periphylla periphylla* (Scyphozoa, Coronatae). Sarsia 84(1): 55–65. 10.1080/00364827.1999.10420451

[B21] Jarms G Morandini AC Silveira FL (2002) Cultivation of polyps and medusae of Coronatae (Cnidaria, Scyphozoa) with a brief review of important characters. Helgoland Marine Research 56(3): 203–210. 10.1007/s10152-002-0113-3

[B22] Katoh K Standley DM (2013) MAFFT Multiple Sequence Alignment Software version 7: improvements in performance and usability. Molecular Biology and Evolution 30(4): 772–780. 10.1093/molbev/mst010 23329690 PMC3603318

[B23] Kearse M Moir R Wilson A Stones-Havas S Cheung M Sturrock S Buxton S Cooper A Markowitz S Duran C Thierer T Ashton B Meintjes P Drummond A (2012) Geneious Basic: an integrated and extendable desktop software platform for the organization and analysis of sequence data. Bioinformatics 28(12): 1647–1649. 10.1093/bioinformatics/bts199 22543367 PMC3371832

[B24] Kölliker A (1853) Bericht über einige im Herbst 1852 in Messina angestellte vergleichendanatomische Untersuchungen. 11. Zeitschrift für wissenschaftliche Zoologie 4: 299–370.

[B25] Kramp PL (1961) Synopsis of the Medusae of the world. Journal of the Marine Biological Association of the UK 40: 7–469. 10.1017/S0025315400007347

[B26] Kumar S Stecher G Li M Knyaz C Tamura K (2018) MEGA X: Molecular Evolutionary Genetics Analysis across Computing Platforms. Molecular Biology and Evolution 35(6): 1547–1549. 10.1093/molbev/msy096 29722887 PMC5967553

[B27] Maas O (1903) Die Scyphomedusen der Siboga-Expedition. Siboga-Expeditie 11. Buchhandlung und Druckerei vormals E. J. Brill, Leiden, 18–23.

[B28] Mariscal RN (1974) Nematocysts. In: Muscatine L Lenhoff HM (Eds) Coelenterate Biology: Reviews and New Perspectives.Academic Press, New York, 129–178. 10.1016/B978-0-12-512150-7.50008-6

[B29] Molinari CG Morandini AC (2019) Update on benthic scyphozoans from the Brazilian coast (Cnidaria: Scyphozoa: Coronatae). Revista Brasileira de Zoociências 20(2): 1–14. 10.34019/2596-3325.2019.v20.27610

[B30] Morandini AC Jarms G (2005) New combinations for two coronate polyp species (Atorellidae and Nausithoidae, Coronatae, Scyphozoa, Cnidaria). Contributions to Zoology 74(1–2): 117–123. 10.1163/18759866-0740102008

[B31] Morandini AC Jarms G (2010) Identification of coronate polyps from the Arctic Ocean: *Nausithoe werneri* Jarms, 1990 (Cnidaria, Scyphozoa, Coronatae), with notes on its biology. Steenstrupia 32(1): 69–77.

[B32] Morandini AC Jarms G (2012) Discovery and redescription of type material of *Nausithoe simplex* (Kirkpatrick, 1890), comb. nov. (Cnidaria: Scyphozoa: Coronatae: Nausithoidae) from the North Atlantic. Zootaxa 3320: 61–68. 10.11646/zootaxa.3320.1.5

[B33] Morandini AC Silveira FL (2001a) New observations and new record of *Nausithoe aurea* (Scyphozoa, Coronatae). Papéis Avulsos de Zoologia 41(27): 519–527.

[B34] Morandini AC Silveira FL (2001b) Sexual reproduction of *Nausithoe aurea* (Scyphozoa, Coronatae). Gametogenesis, egg release, embryonic development, and gastrulation. Scientia Marina 65(2): 139–149. 10.3989/scimar.2001.65n2139

[B35] Müller F (1861) Ueber die systematische Stellung der Charybdeiden. Archiv für Naturgeschichte 27(1): 302–311.

[B36] Neumann-Leitão S Sant’anna EME Gusmão LMO Do Nascimento-Vieira DA Paranaguá MN Schwamborn R (2008) Diversity and distribution of the mesozooplankton in the tropical Southwestern Atlantic. Journal of Plankton Research 30(7): 795–805. 10.1093/plankt/fbn040

[B37] Östman C (2000) A guideline to nematocyst nomenclature and classification, and some notes on the systematic value of nematocysts. Scientia Marina 64(1): 31–46. 10.3989/scimar.2000.64s131

[B38] Russell FS (1956) On the Scyphomedusae *Nausithoë atlantica* Broch and *Nausithoë globifera* Broch. Journal of the Marine Biological Association of the UK 35(2): 363–370. 10.1017/S0025315400010195

[B39] Russell FS (1970) Nausithoeïdae. In: Russell FS (Ed.) The Medusae of the British Isles II.Pelagic Scyphozoa with a Supplement to the First Volume on Hydromedusae. Cambridge University Press, London, 29–37.

[B40] Silveira FL Morandini AC (1997) *Nausithoe aurea* n. sp. (Scyphozoa, Coronatae, Nausithoidae), a species with two pathways of reproduction after strobilation: sexual and asexual. Contributions to Zoology 66(4): 235–246. 10.1163/26660644-06604004

[B41] Silveira FL Jarms G Morandini AC (2003) Experiments in nature and laboratory observations with *Nausithoe aurea* (Scyphozoa: Coronatae) support the concept of perennation by tissue saving and confirm dormancy. Biota Neotropica 2(2): 1–25. 10.1590/S1676-06032002000200009

[B42] Uriz MJ Rosell D Maldonado M (1992) Parasitism, commensalism or mutualism? The case of Scyphozoa (Coronatae) and horny sponges. Marine Ecology Progress Series 81: 247–255. 10.3354/meps081247

[B43] Vanhöffen E (1892) Die Akalephen der Plankton-Expedition. Ergebnisse der Plankton-Expedition der Humboldt-Stiftung 2: 3–28.

[B44] van Walraven L van Bleijswijk J van der Veer HW (2020) . Here are the polyps: *in situ* observations of jellyfish polyps and podocysts on bivalve shells. PeerJ 8: e9260. 10.7717/peerj.9260 PMC7261476 32523816

[B45] Werner B (1970) Contribution to the evolution in the genus *Stephanoscyphus* (ScyphozoaCoronatae) and ecology and regeneration qualities of *Stephanoscyphus racemosus* Komai. Publications of the Seto Marine Biological Laboratory 18(1): 1–20. 10.5134/175623

[B46] Werner B (1971) *Stephanoscyphus planulophorus* n. spec., ein neuer Scyphopolyp mit einem neuen Entwicklungsmodus. Helgoländer wissenschaftliche Meeresuntersuchungen 22: 120–140. 10.1007/BF01611366

[B47] Werner B (1979) Coloniality in the Scyphozoa: Cnidaria. In: Larwood G Rosen BR (Eds) Biology and Systematics of Colonial Organisms.Academic Press, London, 81–103.

